# No decision about me, without me: Collaborating with young people in mental health research

**DOI:** 10.1002/jcv2.12291

**Published:** 2024-12-11

**Authors:** Alex Lloyd, Tom (Chin‐Han) Wu, Laura Lucas, Adeola Agunbiade, Romana Saleh, Pasco Fearon, Essi Viding

**Affiliations:** ^1^ Clinical, Educational and Health Psychology, Psychology and Language Sciences University College London London UK; ^2^ Young People's Advisory Group London UK; ^3^ Centre for Family Research Department of Psychology University of Cambridge Cambridge UK

**Keywords:** co‐design, co‐production, developmental psychopathology, participatory research, PPI

## Abstract

Involving young people with lived experience in youth mental health research is important. In recognition of the value of collaborating with experts by experience, international funders are increasingly mandating that mental health research is developed by teams that include individuals from the population of study. Yet, research into how Patient Public Involvement, specifically co‐production and co‐design, is implemented in youth mental health research is limited to date. The current review examined this question and identified common practices for collaborating with experts by experience in young people's mental health research. Academic databases were systematically searched for studies that had involved young people in mental health research, had described these activities, and had reported some demographic information about the experts by experience. From a total of 2130 studies that were screened, 37 studies were eligible for inclusion. The use of co‐production and co‐design spanned a wide range of topics, including interventions, digital support tools and psychometric studies. Interactive workshops were the primary method of engaging experts by experience, although some studies utilised interviews or focus groups. From the reviewed studies we identified common methodological practices including: Scene setting, utilising a cyclical process, ensuring appropriate engagement and recognition of the cultural context. We draw on these findings to suggest common methods for conducting co‐production and co‐design activities, and emphasise the importance of ensuring experts by experience are respected and safeguarded throughout their collaboration in research. We also outline areas that deserve future attention and development, and include a response from two young people aged 16–18 and their suggestions for improving and extending co‐production methods.

## Graphical abstract

Involving young people with lived experience in youth mental health research is important. The current review examined this question and identified common practices for collaborating with experts by experience in young people's mental health research. From the reviewed studies we identified common methodological practices including: Scene setting, utilising a cyclical process, ensuring appropriate engagement and recognition of the cultural context. We draw on these findings to suggest common methods for conducting co‐production and co‐design activities, and emphasise the importance of ensuring experts by experience are respected and safeguarded throughout their collaboration in research.

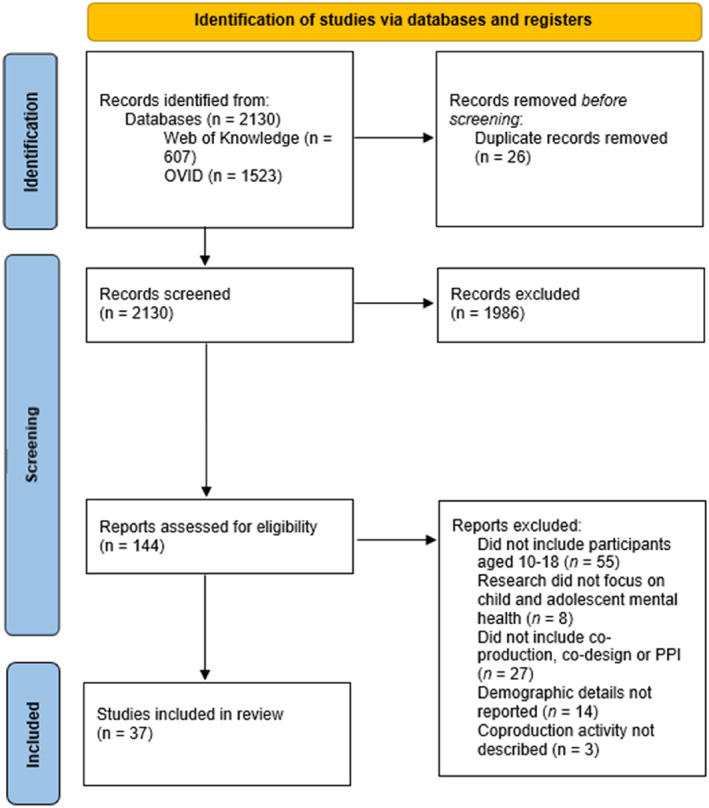

**Key points**

- Involving individuals with lived experience with mental health problems in research is important, but there is limited information about common practices used in Patient Public Involvement (PPI) methods, such as co‐production and co‐design.
- We review studies using PPI methods, including co‐production and co‐design, finding common methods based around the themes of: Scene setting, cyclical engagement, appropriate engagement and cultural context.
- PPI methods can be improved to ensure experts by experience are safeguarded during their collaboration, are encouraged to engage in research using collaborative methods, are appropriately remunerated, and represent the diversity of populations that experience mental health problems.

## INTRODUCTION

Involving the views of individuals with lived experience in mental health research is important and international funders are increasingly mandating that mental health research is developed by teams that include individuals from the population of study. For example, the Wellcome Trust, the UK Research and Innovation (UKRI) and the National Institute for Health and Care Research (NIHR) all stipulate that research projects should include co‐production or co‐design with experts by experience (Farr et al., 2021; NIHR, 2020; UKRI, 2022). Despite the recognised importance of embedding the views of experts by experience within research on young people's mental health (e.g., Foulkes & Stapley, 2022; Whitmore & Mills, 2022), we do not currently have a clear overview of how co‐production, co‐design are typically implemented within the research cycle. Here, we define experts by experience as individuals with knowledge of a mental health topic due to having lived with psychopathology, or used mental health services, relevant to the subject (Happell et al., 2023). Unlike academic experts, whose experience comes from their training, experts by experience have gained their understanding through personal experience relevant to the mental health problem being studied. The current paper will review how PPI, specifically co‐production and co‐design, have commonly been implemented in mental health research involving young people.

Mental health research is costly in terms of both fiscal and human resources (MQ Mental Health, 2019; Woelbert et al., 2021) and including those with lived experience in the research process can be vital for informing research and implementation questions. In the case of developmental psychopathology research, we cannot assume that the same testing protocols or interventions that work for adults are necessarily suited for young people without adaptations. As an example, a recent large‐scale mindfulness intervention trial delivered in schools (Kuyken et al., 2022) failed to find improvement in mood disorder symptoms of young people, despite mindfulness interventions being effective for treating mood disorders in adults (e.g., Reangsing et al., 2021). The authors speculated that the school setting and universal participation may not have been optimal for all young people and that a co‐design element to adapt the intervention to the school setting could have improved the intervention (Montero‐Marin et al., 2022). To maximise the benefits of mental health research, it is important to ask young people what they find engaging, acceptable and meaningful. Such questions, and others that are vital to the success of mental health research, can be answered by working alongside experts by experience (Foulkes & Stapley, 2022).

Collaborating with experts by experience is referred to Patient and Public Involvement (PPI) in research (Perowne et al., 2024). Patient Public Involvement is an umbrella term that includes a range of approaches to collaborating with experts by experience, with different levels of power sharing between researchers and experts by experience. For example, PPI can refer to projects where experts by experience are consulted on a project, but do not have the ability to make decisions about the study. In contrast, PPI methods such as co‐production and co‐design often assign greater responsibility to experts by experience, meaning they have greater power to implement change within a research project. Co‐production, for example, is an approach to PPI in which experts by experience are equitable partners within a project and often collaborate throughout the research cycle (e.g., Pavarini et al., 2019). Experts by experience are similarly considered equitable partners in co‐design, though these activities are typically restricted to the development of interventions, services or products (e.g., Bevan Jones et al., 2020).

One framework for understanding how the views of experts by experience can be embedded in research is the ‘ladder of participation’ (Hart, 1992). Derived from a theory of civic participation (Arnstein, 1969), the ladder of participation identifies the extent to which the intended users of a service or intervention can engage in decision‐making related to their participation. At the bottom of the ladder, individuals with lived experience have minimal participation and are passive recipients of decisions made by those who, relationally, hold positions of power (e.g., academic researchers, clinicians, or service providers). At the top of the ladder are activities initiated by individuals from the population, referred to as ‘citizen control’ (Arnstein, 1969). In developmental psychopathology, citizen control would involve young people directing their own research into mental health and examples of this type of activity are limited within this field. Patient Public Involvement, including co‐production and co‐design, can occur at different points along the ladder of participation. For example, research that is initiated by adults but in which young people have shared decision‐making about the design of research materials would qualify as PPI though this form of participation is found lower on the ladder (see Sellars et al., 2021; Siston et al., 2023). The ladder of participation can be utilised to identify how decisions are made within a project and whether the relationships between academic researchers and those with lived experience are equal and reciprocal (for one example, see Funk et al., 2012). Although the ladder of participation can be helpful when considering different types of involvement within mental health research, the tool has been critiqued insofar as it assumes participation is exclusively positive and does not recognise unintended and possibly negative consequences from greater involvement in research (Cahill & Dadvand, 2018). Furthermore, the ladder could be taken to suggest ‘better’ and ‘worse’ ways of PPI and that every research project should strive for ‘citizen control’ in all aspects of the study. This may not be appropriate, or possible, for example, given the expertise and training required to design certain aspects of study protocol. In response to these critiques, other models have been proposed that recognise the complexity of the relationship between researchers and experts by experience (e.g., the 7P Model; Cahill & Dadvand, 2018).

There are several existing resources that discuss how to work alongside experts by experience within research. For example, the NIHR highlights that sharing power in co‐production may involve deciding which parties are involved in which decisions, rather than all stakeholders being expected to contribute to every decision (NIHR, 2020). Indeed, transparency in how decisions are made, and by whom, is crucial for collaborating with experts by experience in research while respecting the relative expertise of each group (McPin Foundation, n.d.). Yet, to our knowledge, there is limited guidance on how best to conduct co‐production and co‐design with adolescents. This absence is notable, as power imbalances that may already exist between researchers and experts by experience may be further compounded by ‘invisible’ dynamics when working with adolescents, such as age, financial or educational dynamics (Perera, 2018).

Alongside benefits to academics and their research, embedding co‐production or co‐design within mental health research also has demonstrable benefits for the young people who collaborate on these projects. Involvement in the co‐production or co‐design of research can empower young people to engage with research methodologies (Campbell et al., 2019), provide educational value (Watson et al., 2023; Whitmore & Mills, 2022), develop their self‐confidence (Foulkes & Stapley, 2022), self‐efficacy (Hawke et al., 2020), and allow them to meet new people (Pavarini et al., 2019).

Identifying and developing common methods for implementing PPI methods such as co‐production or co‐design in mental health research is also important in the context of the Open Science movement. Open Science refers to the commitment to make research practices and data openly available to researchers and the wider community (Nosek et al., 2015). To ensure that co‐production and co‐design can be transparently described as part of the research process, it is valuable to establish frameworks or a taxonomy that capture the range of methodologies currently used in co‐production and co‐design (Bergin et al., 2020). In doing so, best practices can be supported, and research outputs can be critically evaluated in relation to the nature of quality of PPI work undertaken.

A further reason to identify common methods used in co‐production and co‐design is to establish a taxonomy of methodological tools that can be used by researchers seeking to adopt co‐production and co‐design methods in their research. In addition, by identifying methods currently used, this review may also facilitate focus on areas that deserve further attention and development. Establishing common methods in this field will ensure that experts by experience are more likely to have a consistent experience when collaborating in mental health research, as well as having a benchmark with which to compare their experience. As co‐production and co‐design methodologies do not necessarily require approval from an ethics review board (Co‐Production Collective, 2022), establishing a basic methodological benchmark through identifying common practices will ensure that researchers can be accountable to a minimum standard when designing, conducting and reporting PPI work with young people.

Here we conduct a scoping review on how PPI, including co‐production and co‐design, have been implemented in developmental psychopathology research—that is, mental health research that involves young people. Given the relative dearth of literature in this field, we did not consider a systematic review the most appropriate evidence synthesis method. Rather, we conducted a scoping review to examine how PPI methods are utilised in developmental psychopathology and identify gaps in this methodology (Munn et al., 2018). Using this approach, we draw on the existing literature to suggest common practice methods for conducting co‐production and co‐design activities to integrate the views of young people into mental health research in a robust, methodologically rigorous manner. Such development work may also support future endeavours to conduct more precise syntheses of evidence in this field, such as systematic reviews (Munn et al., 2018). We also outline areas that deserve future attention and development and include a response from two young people aged 16–18 (authors RS and AA), with their suggestions for improving and extending co‐production and co‐design methods. The objective of this review is to identify common methods currently used when conducting co‐production and co‐design with young people to study developmental psychopathology. Further, a secondary objective is to review common reporting standards used to describe this methodology in published research.

## METHODOLOGY

### Eligibility criteria

Studies were deemed suitable for inclusion if they meet the following criteria:

1. Included participants aged between 10 and 18.
2. Conducted research on children and young people's mental health.
3. Included co‐production or co‐design.
4. Reported demographic characteristics of the experts by experience. Specifically, studies were required to include a minimum of information about the age of experts by experience, to allow us to compare studies against criterion 1.
5. Provided a description of at least one PPI activity.
6. Was available in English language and published in a peer‐reviewed journal.

The decision to include a minimum age for eligible studies was based on a soft search, which did not identify any studies that recruited experts by experience with aged below 10. We opted to only include studies that reported information relevant to the eligibility criteria in the manuscript, rather than contacting authors to clarify additional details (e.g., demographic information of experts by experience of remuneration provided). The rationale for this decision was based on the framing of the review in context of the Open Science Movement; specifically, we aimed to review studies based on the information they reported in text that would allow independent researchers to replicate their method. Therefore, authors of studies identified during the screening phase were not contacted for information relevant to the eligibility criteria.

### Search strategy

**FIGURE 1:**
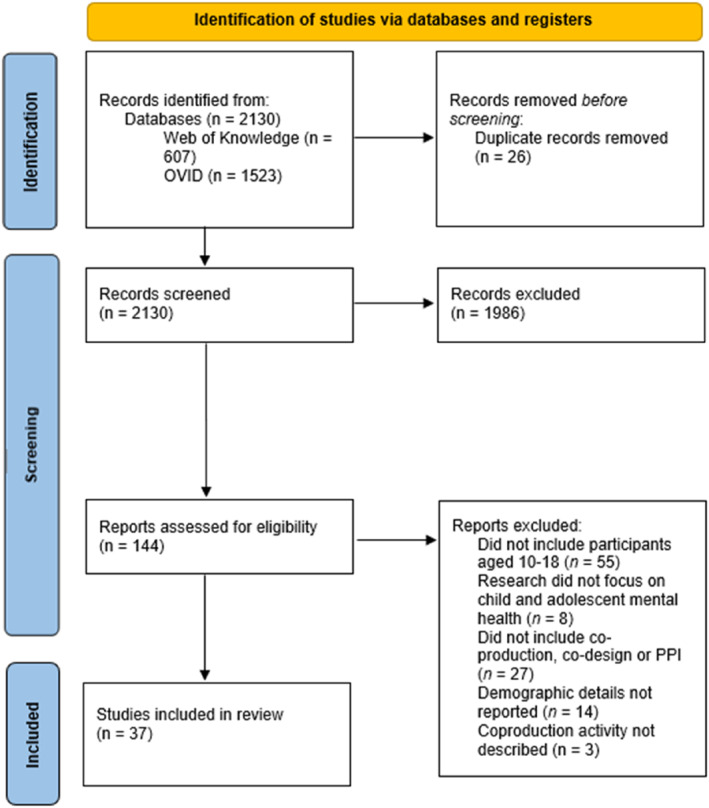
PRISMA chart outlining the number of studies excluded after the initial search and screening procedure.

We conducted searches of EMBASE, MEDLINE and PsycINFO (using the OVID interface), and Web of Knowledge. We did not opt to search grey literature for research conducted in community or charity settings as one of the aims of this review was to consider how PPI is reported in academic research. However, we point anyone interested in PPI in non‐academic research to Robotham et al. (2023) and Peer Power (2021). We completed the searches of the academic databases between May and June 2023. The search term we utilised was derived from the Patient, Intervention, Comparison and Outcome (PICO) framework. However, as our review does not include a comparison intervention, our search only included terms for patients, ‘intervention’ (i.e., use of co‐production or co‐design), and outcomes. The search terms we used was: adolesc* OR child* AND psychiatry OR psychopatholog* OR mental health AND PPI OR co‐produc* OR co‐design*. We include the phrase child* in the search criteria as some studies conduct PPI with adolescents on the topic of child mental health. The reference lists of included publication were also searched. See Figure 1 for a PRISMA flow chart outlining the number of articles excluded after initial screening. As traditional tools for assessing research quality (e.g., the Assessment tool for cross sectional studies; Downes et al., 2016) include criteria that are not relevant to PPI methods (e.g., criteria to establish statistical significance), we did not include a formal assessment of quality of each included paper.

### Study selection and data extraction

Identified studies were screened against the eligibility criteria agreed by the first author (AL). In cases where there was uncertainty about the eligibility of a study, the authors discussed the issue and reached a consensus about whether the study was suitable for inclusion. An additional two authors (TW and LL) examined 10 studies each (five that were considered eligible to be included and five that were considered ineligible; 13.89% of the total number of reports assessed for eligibility) against the eligibility criteria to reduce bias in the screening procedure. Where there was disagreement, the authors met together to reach a consensus about whether the study met the inclusion criteria, which happened in the case of five studies. Once the eligibility of studies had been determined, AL then extracted the following data from eligible studies into a Microsoft excel file:

- Study characteristics: Authors list, year of publication, country, design of the co‐production, co‐design or PPI activities, recruitment method for experts by experience, inclusion and exclusion criteria for experts by experience, topic of study and sample size.
- Population details: Age, gender, ethnicity, socioeconomic information, and expertise of those recruited.
- Co‐production, co‐design or PPI characteristics: Description of the activity, stage of the research cycle the experts by experience collaborated on, number of sessions, length of sessions, output or impact of the collaboration, training and supervision offered, and remuneration received.

### Data synthesis

As the outcome of interest was the operationalisation of PPI, including as co‐production and co‐design, within the included studies, we did not conduct any quantitative analysis. Further, as there was significant variability in the level of detail reported in the included studies, we were unable to conduct a qualitative synthesis of the methods used in included studies. Rather, results were synthesised in a narrative review which allowed us to identify common themes in the methods used to conduct the PPI sessions. Specifically, we synthesised findings by identifying the methods that were most commonly reported by authors. We synthesised findings while taking an neutral approach to the effectiveness of these methods, which allowed us to consider the advantages and disadvantages of commonly used methods, as well as how the effectiveness of these approaches may differ between populations of experts by experience. Through this approach to data synthesis, we were able to address the aims of this review to identify common methods used in co‐production and co‐design, consider how these methods are reported, and identify avenues for future research to improve PPI methods.

## RESULTS

### Summary of studies

#### Demographic variables and study characteristics

Thirty‐seven studies met our inclusion criteria. These studies included a total of 1383 PPI contributors, though the sample sizes within each study varied greatly (ranging from 4 to 134). The majority of included studies exclusively reported results from their PPI activities (*N* = 23), whereas the remaining 14 studies were research reports that also detailed results from PPI work. The topics of study varied across the manuscripts and included research on specific mental health problems (*N* = 16), intervention development (*N* = 12), service development (*N* = 4) and psychometric research (*N* = 1). Within these studies six focused on anxiety, four focused on depression, one focused on conduct problems, one focused on eating disorders and one focused on psychosis, whereas the remaining studies focused on general mental health, help‐seeking or service provision. The reporting on demographic variables was variable and a common limitation identified in most studies was that experts by experience involved in the research were not representative of the wider population (See Supplemental Information I and Supplementary Table 1 for information about the demographic features of experts by experience in the included studies, as well as the topics studied using these methods). Studies were conducted in a range of countries, though we note these were predominantly from the Global North and included Australia (*N* = 10), Canada (*N* = 2), India (*N* = 1), Indonesia (*N* = 1). Ireland (*N* = 2), Italy (*N* = 1), New Zealand (*N* = 1), the UK (*N* = 17) and the USA (*N* = 1). Only one study was conducted in multiple countries for the same study (specifically Spain, Italy, Denmark and Iceland).

#### Implementation of co‐production, co‐design or patient public involvement

All included studies utilised either interviews and focus groups (*N* = 14) or workshops (*N* = 24) to collaborate with experts by experience. Interviews and focus groups were utilised to solicit young people's views on the topic of study, whereas workshops were typically utilised for broader purposes, including developing stimuli, providing feedback on study designs, and reviewing findings from studies. For example, several studies utilised workshops to create narratives that were introduced into interventions for psychopathology during the development stage (Abel et al., 2020; Christie et al., 2019; Gabrielli et al., 2020; Gellatly et al., 2019; Li et al., 2022; Povey et al., 2020; Syed Sheriff et al., 2022). Experts by experience collaborated across the research cycle and in some cases, across multiple stages of the research cycle within the same study (Bennett et al., 2020a, 2020b; Davison et al., 2022; Gellatly et al., 2019; Hill et al., 2022; Li et al., 2022; Mindel et al., 2022; Neill et al., 2022; Povey et al., 2020). Twenty‐seven studies recruited experts by experience in the initial design of the study, and seven studies asked experts by experience to feedback on content or findings from their research (see Supplementary Table 1). Only four studies (Bennett et al., 2020a, 2020b; Mindel et al., 2022; Thomson et al., 2022) asked experts by experience to collaborate on analysing or interpreting findings of the research.

Co‐production and co‐design were used to inform the study of young people's mental health across a range of research areas. Collaborating with experts by experience led to important adaptations to these studies, with the aim of increasing the likelihood they will be acceptable and beneficial to individuals in the target population. From the methods described by the included studies, we identified common themes in the methods used to conduct co‐production or co‐design with adolescents, which we describe below.

### Scene setting

Across interviews, focus groups and workshops, researchers described a period of ‘scene setting’, whereby experts by experience were provided with information about the project. This process included important information regarding informed consent, confidentiality, and safeguarding procedures (Davison et al., 2022; Edridge et al., 2018; Hill et al., 2022; Li et al., 2022; Moltrecht et al., 2022; Morote et al., 2022). Scene setting was used to emphasise and clarify the contribution of these individuals and explain that they had unique knowledge about the topic, as experts in their own experience, which could directly benefit the research project (Culbong et al., 2022; Moltrecht et al., 2022; Morote et al., 2022). This explanation provided context for the role of the experts by experience, helping to identify them as equal contributors in the co‐production or co‐design activities (Cheng et al., 2021; Moltrecht et al., 2022; Morote et al., 2022).

While scene setting, researchers also outlined the aims of the PPI activity and the desired outcomes from collaborating with the experts by experience (Morote et al., 2022). Through outlining these aims, researchers were able to focus the session on elements of the project that the experts by experience were able to contribute to, with the aim of maximising the usefulness of the sessions (Cheng et al., 2021; Moltrecht et al., 2022; Morote et al., 2022). This suggests the importance of identifying features of the project that experts by experience can contribute to a priori.

### Ensuring appropriate engagement

Several studies reported considering how to collaborate with experts by experience while ensuring they were safeguarded from risks that could arise through their role. Due to the topics of study, many of the experts by experience had current or previous experience of mental health problems (including anxiety, depression, or psychosis; Bennett et al., 2022a; Edridge et al., 2018; Latif et al., 2017; Libon et al., 2023; Realpe et al., 2020), which raises ethical considerations when working with potentially vulnerable populations (Lloyd‐Richardson et al., 2015). To mitigate any such risks to participating experts by experience, several studies utilised creative workshops, most commonly when co‐designing research. Creative methods were deemed a less direct method of discussing the topic, helping to avoid inducing negative affect in experts by experience or creating conditions where experts by experience were expected, or might feel pressurised, to disclose personal information (Povey et al., 2022). For example, Brooks and colleagues (2021a) used activities such as drawing, model making, and sculpting to elicit young people's views on proposed content for a novel intervention to improve mental health literacy in Indonesia without requiring young people to disclose information about their own experiences of mental health problems.

### Cyclical processes

Twenty‐six out of 37 studies conducted more than one co‐production or co‐design session with experts by experience (Bennett et al., 2022a, 2022b; Björling et al., 2019; Brooks et al., 2021a, 2021b; Cheng et al., 2021; Christiea et al., 2019; Culbong et al., 2022; Davison et al., 2022; Gellatly et al., 2019; Gobat et al., 2021; Gonsalves et al., 2019; Hackett et al., 2018; Hill et al., 2022; Hugh‐Jones et al., 2022, 2023; Li et al., 2022; Libon et al., 2023; Mindel et al., 2022; Moltrecht et al., 2022; O’Brien et al., 2022; Povey et al., 2020; Povey et al., 2022; Realpe et al., 2020; Syed Sheriff et al., 2022; Stoyanov et al., 2021; Thomson et al., 2022; Thorn et al., 2020; Zieschank et al., 2021). These studies reported that their collaborations with experts by experience were conducted in an iterative manner, whereby feedback from sessions were used to inform the design of the study, which was subsequently presented to experts by experience again for further feedback (Bennett et al., 2022a, 2022b; Davison et al., 2022; Gobat et al., 2021; Povey et al., 2020). Using this iterative, cyclical process ensured the views of experts by experience were embedded throughout the project and allowed their ideas to be further refined, compared to studies where only single PPI sessions were utilised (Davison et al., 2022; Zieschank et al., 2021). Notably, studies that had several co‐production or co‐design sessions did not necessarily collaborate with the same experts by experience in each session, though it was unclear whether this was by design, due to attrition, or a practical consideration from the researchers (Björling et al., 2019; Cheng et al., 2021; Davison et al., 2022; Gobat et al., 2021; Hackett et al., 2018; Povey et al., 2020; Realpe et al., 2020; Thorn et al., 2020).

### Cultural context

There were some notable examples of studies that explicitly addressed and embedded the culture of experts by experience within their co‐production or co‐design activities. For example, Culbong et al. (2022) conducted ‘yarns’ with Aboriginal experts by experience, a method of communication used by Aboriginal communities to facilitate discussion. This technique utilises storytelling to draw out views on topics that individuals may not want to directly disclose, with researchers also sharing some information about themselves to establish a reciprocal relationship between individuals involved in the yarn (Bessarab & Ng'Andu, 2010). Through utilising this culturally appropriate method, the researchers were able to elicit views from the experts by experience about mental health service access and provision that are not typically discussed by Aboriginal young people (Culbong et al., 2022). Several other studies also reported adapting the co‐production or co‐design activities to the cultural context of the experts by experience (Brooks et al., 2021a; Christiea et al., 2019; Gonsalves et al., 2019; Povey et al., 2022). Through collaborating with experts by experience using methods that were more familiar to them, researchers aimed to establish better working partnerships and elicit more authentic views from these individuals (Brooks et al., 2021a; Culbong et al., 2022; Gonsalves et al., 2019; Povey et al., 2022). Indeed, adapting PPI activities to the cultural context of experts by experience may be one method to improve the representativeness of experts by experience in mental health research.

### Impact of co‐production and co‐design

Across the studies reviewed, experts by experience made a significant impact as collaborators. In studies developing a new mental health intervention, the involvement of experts by experience meant that fundamental changes were made to the design of these interventions to make them more relatable (Björling et al., 2019; Gabriell et al., 2020; Gonsalves et al., 2019), engaging (Grové, 2021; Hill et al., 2022; Hugh‐Jones et al., 2022, 2023; O’Brien et al., 2022; Povey et al., 2020, 2022; Realpe et al., 2020; Stoyanov et al., 2021), and accessible (Hill et al., 2022; Moltrecht et al., 2022; O’Brien et al., 2022) to young people. For example, Realpe and colleagues (2020) based the aesthetic design for their phone‐based application on input from experts by experience and added features such as contact details for emergency services. Notably, adjustments made across different studies removed barriers that may have prevented young people from engaging with these novel interventions (Realpe et al., 2020), which can impact their efficacy (Davison et al., 2022). By involving young people from a wide range of communities, interventions were tailored for young people from diverse backgrounds, including those from cultures where there is social stigma around mental health (Gonsalves et al., 2019). This finding highlights the importance of co‐production approaches for developing study protocols for populations that are typically underrepresented in mental health research (i.e., Western, educated, industrialised, rich and democratic (WEIRD) populations).

Other notable examples of the impact of collaborating with experts by experience included these individuals helping to identify themes from qualitative interviews (Bennett et al., 2022a), interpreting the findings of qualitative interviews (Bennett et al., 2022b), or developing age‐appropriate stimuli in the creation and validation of new scales (Davison et al., 2022). Several studies also collaborated with experts by experience to inform the delivery of mental health care, highlighting barriers and facilitators to the provision of these services to general populations of adolescents (Brooks et al., 2021b; Hackett et al., 2018) and those from minority ethnic groups (Culbong et al., 2022).

## DISCUSSION

The current review summarised common methodologies used in co‐production and co‐design with young people in mental health research. We have highlighted that scene setting, ensuring appropriate engagement, using a cyclical process, and understanding the cultural context of experts by experience are commonly used methods for collaborating with young people in mental health research. These methods allowed researchers to form meaningful collaborations with experts by experience and use their views to improve the acceptability of research to the target population. However, our review also highlighted significant limitations in current practices, including a lack of representativeness in experts by experience, a lack of training provided to experts by experience, and a lack of remuneration for their collaboration. Further development of co‐production and co‐design methods are required to address these points to ensure that young people can engage as meaningful partners in mental health research.

Despite the limitations of existing studies, we can draw on these findings to suggest a number of common practice methods for conducting PPI, such as co‐production or co‐design, activities. These recommendations are based on exemplars of good practice identified in the reviewed studies. Specifically, these recommendations were based on reflections from researchers in which the rationale for their co‐production or co‐design activities were justified. We therefore report these recommendations as methodological details that should be reported in future research when describing co‐production and co‐design in mental health studies. Further, we encourage researchers to consider the appropriateness of these approaches for specific populations of experts by experience:

1. *Scene setting.* Scene setting should be utilised to provide experts by experience with important information about their role as collaborators including areas of the project that they can contribute toward and the time commitment involved (i.e., single or multiple sessions). Setting the scene to explain how experts by experience can inform the project also recognises that there are some aspects of research that experts by experience may not be able to contribute toward. For example, it may be inappropriate to expect experts by experience to have sufficient knowledge about statistical analyses to contribute to decisions about how to analyse and interpret the outcomes of quantitative research, as such knowledge requires training often outside the scope of co‐production activities. However, it is important to note that some studies delivered training to experts by experience to mitigate these issues: Bennett et al. (2022a, 2022b) trained experts by experience in qualitative data analysis, allowing them to share decision‐making about how the findings of the study were analysed.
2. *Safeguarding.* The extent and nature of the collaboration should be carefully considered and experts by experience safeguarded throughout the collaboration. It is important for researchers to monitor the wellbeing of these individuals throughout their role, recognising that these methods can elicit negative feelings for experts by experience (Pavarini et al., 2021). For example, one study recruiting adolescents with lived experience of suicidal ideation reported that some individuals reported feeling suicidal because of their participation in the workshop (Thorn et al., 2020). As PPI, such as co‐production and co‐design, do not qualify as formal research participation, this methodology often does not require institutional ethical review (see Co‐Production Collective, 2023). Therefore, there is a heightened need for robust safeguarding procedures to be established within research teams before co‐production and co‐design is employed with vulnerable young people. Such safeguarding procedures will need to account for whether experts by experience are involved in co‐producing research, in which case ongoing monitoring over multiple sessions may be appropriate, vs. single co‐design sessions where extensive debriefing may instead be required.
3. *Sensitivity and care in selecting experts by experience.* It is important to weigh up who may most appropriately contribute to a particular research project. For example, one study working with experts by experience to improve mental health service provision for Aboriginal young people specifically recruited individuals who were not active clients of the service to avoid potential conflicts of interest (Culbong et al., 2022). However, this does not preclude collaborating with experts by experience who are current users of the service being examined, as several studies restricted their inclusion criteria to current service users because they had the most expertise on the study topic (Edridge et al., 2018; Hackett et al., 2018; Latif et al., 2017; Realpe et al., 2020). A further consideration when recruiting experts by experience is the need to actively include individuals from underserved communities. For example, this may involve specifically recruiting individuals from minority ethnic groups, care experienced young people, or those with language and communication disorders, as these individuals are typically underrepresented in mental health research.
4. *Degree and variety of engagement.* Researchers should weigh up whether to work with the same group of experts by experience or separate groups across the project. Collaborating with different groups of experts by experience may be beneficial for integrating diverse views. However, working with the same group of experts by experience can provide an opportunity for ideas to be further refined (Davison et al., 2022) and produce stronger collaborations as experts become more familiar with the research process (Bennett et al., 2022a, 2022b; Thomson et al., 2022). Further, collaborating with the same group of experts by experience can provide opportunities for researchers to demonstrate how views of experts by experience have been integrated into the project (Davison et al., 2022), thus emphasising their role as equal partners within the research process. An ideal approach to co‐production and co‐design would strive for a diverse set of experts by experience from the outset and cultivate a relationship of trust and mutual collaboration over time, though co‐design methods may incur less involvement compared to co‐producing research.
5. *Fair remuneration for work undertaken.* To create a fair and respectful co‐working environment, the experts by experience should be appropriately remunerated for their contribution. Most studies included in the review (*N* = 21) did not report whether experts by experience were remunerated for their role. This does not, however, mean that experts by experience were unpaid in these studies, as we did not contact authors about information that was not reported in their manuscripts. Nevertheless, a lack of financial compensation can act as a barrier to maintaining participation and may contribute to the lack of representativeness in experts by experience. However, it is important to ensure that financial compensation does not introduce coercive power dynamics between researchers and experts by experience. For example, the introduction of monetary compensation may lead some experts by experience to feel that they are required to agree with the researchers or risk losing their payment (Perera, 2018). This issue can be mitigated through explaining to experts by experience that their genuine views are being sought and disagreement will not affect their remuneration.

Our recommendations are based on common practices currently employed when conducting co‐production and co‐design with young people. This review contributes to this literature by providing guidance for researchers planning on using these methodologies, along with avenues to improve PPI methods. We acknowledge that some of the suggestions we have raised have been identified by other organisations using PPI methods. For example, the Co‐Production Collective (2022) provide guidance on remunerating experts by experience and the McPin Foundation has several resources for conducting co‐production (https://mcpin.org/resources/). We suggest that improved transparency in reporting standards around the use of co‐production will better allow for these methods to be audited and improved upon, as some studies included in the review did not report key information about the experts by experience in the studies included in this review (see Supplementary Table 1). Future research could, for example, establish quality assessment tools for PPI methods similar to other tools used to assess research quality (e.g., the Appraisal tool for cross sectional research; Downes et al., 2016).

In addition to these methodological developments, future research should also evaluate whether experts by experience meet the criteria to be authors on journal articles. A minority of studies included in the current review also included experts by experience as authors on academic publications (see Supplementary Information). According to some guidelines, such as those of the American Psychological Association (American Psychological Association, 2020), experts by experience may not meet the criteria for co‐authorship (see Bakermans‐Kranenburg & Ijzendoorn, 2024, for a discussion). Yet, guidelines such as the Contributor Roles Taxonomy (CRediT; Brand et al., 2015) detail several areas under which work by experts by experience might qualify them for authorship. These areas include (but are not limited to): Conceptualisation of research goals, methodological contributions to the design of studies, and—given recent emphasis in involving expert by experience in grant applications (Farr et al., 2021; NIHR, 2020; UKRI, 2022)—funding acquisition. These considerations, as well as the preferences of experts by experience, should be carefully managed when determining whether experts by experience meet the criteria for authorship of journal articles.

### Young people's response

Following the review, and drawing on our personal experiences (authors AA and RS), it is notable that scene setting is a valuable tool used in the eight studies (Cheng et al., 2021; Culbong et al., 2022; Davison et al., 2022; Edridge et al., 2018; Hill et al., 2022; Li et al., 2022; Moltrecht et al., 2022; Morote et al., 2022), and it is important that it is used in the future. Scene setting validates experts‐by‐experience, making us feel more equal (which may not be common due to the potential power imbalances between researchers and experts‐by‐experience), which is also a principal value of co‐production. Not only this, but scene setting allows for experts‐by‐experience to fully understand their role, allowing their contributions to be more refined. Interviews, focus groups and workshops are all equally constructive when tailored to the study's aims. Creative methods are beneficial for younger experts‐by‐experience (Brooks et al., 2021a; Povey et al., 2022), though we understand that creative methods may be less effective for older experts‐by‐experience due to limitations in communication of complex topics. In our role as young people's advisors for a recent project we participated in, scene setting by the researcher helped us understand precisely what the project was about, our roles in each stage and how we would be supported. This helped us feel more confident before the meetings because we knew what exactly was expected of us.

Whilst the studies reviewed here made an impressive effort to foster the participation of young people in research, there are certain aspects the studies have done less well. In 27 studies, the experts‐by‐experience were included at the initial study design, however only four studies included the experts by experience in analysis and interpretation of research. The limited involvement in studies means there was not an opportunity for ideas or suggestions made by young people to be clarified by experts‐by‐experience before they were implemented in the research. In our role as a member of the young people advisory group for a recent project, the researcher made sure to include the young people in the interpretation of research. Not only did this help us feel valued, it also made sure the interpretation fully reflected the views of people who will directly benefit from future research.

Additionally, less than half of the studies recorded financial compensation and two studies among this recorded voucher payments (Latif et al., 2017; O’Brien et al., 2022) which are often not the preferred means of payment. It is important to have adequate payment to strengthen the values of co‐production or co‐design and evidence of a sense of equality between the experts‐by‐experience and the researchers. Persons with lived experience play a crucial role in research and should be compensated accordingly for their expertise.

Despite the previous efforts in co‐production and co‐design, there is a lot of work which needs to be done in future research to truly implement the values of the methodology, ethically and accurately. For instance, using the same groups of diverse experts‐by‐experience with a cyclical methodology would help them refine their ideas, make them more comfortable and confident in their opinion while increasing their understanding of the project. Those outcomes are reflected in our own experience of being involved in prior research, where the above methodology was used.

Payment guidance should be developed and shared with experts‐by‐experience before their involvement. Experts by experience need to know exactly what compensation they will receive to help them manage their expectations. Before we began our roles as young people's advisors for a recent research project, we were informed of the compensation we would receive and were able to make the informed decision to participate. This information is important to not only give a sense of equality, but also to prevent putting the experts‐by‐experience in a difficult position. It is also important to ensure paid training should be given for roles that require understanding of technical materials, and researchers should emphasise that such payments and any subsequent remunerations should not stop experts‐by‐experience from giving honest feedback.

Furthermore, safeguarding and ethics should be top priority and experts‐by‐experience should be treated the same as formal research participants. By informing experts‐by‐experience of their rights and that there is a support system available if they need any help, especially for particularly sensitive topics, experts‐by‐experience can and will feel more comfortable in the group and working with the researchers; allowing for both ethics to be completely upheld and for the opinions given by experts‐by‐experience to be more valid.

### Limitations and conclusion

There are some important limitations to consider with the current review. First, the restriction on English‐language publications could mean that we did not identify PPI work being conducted in non‐English speaking settings, such as the Global South. This limitation is notable as it could mean we overlooked more culturally diverse methods used in co‐production and co‐design. A further limitation is that we did not contact authors to enquire about information that was missing from the published manuscripts (e.g., demographic information or remuneration policies). This decision was made as we wanted to review how co‐production and co‐design were reported, as well as omissions to information that was reported that would hinder replicability of these methods in context of the Open Science Movement. Nevertheless, in opting not to contact authors, this review may not capture details in the PPI methods that were not reported in published manuscripts.

In sum, we have reviewed current methods used in co‐production and co‐design in mental health research with young people. The common themes we identified have been used to provide a guide for future researchers conducting PPI. While our review was limited to research on mental health, the methods identified may be informative for other research areas, though additional considerations may be required to tailor these methods to other populations (e.g., Chinn & Pelletier, 2020). This review has demonstrated that there is significant heterogeneity in reporting standards for PPI. This heterogeneity is particularly notable in context of the recognised importance of Open Science and the need for research to be reported transparently. We have suggested improvements that future research can make to co‐production and co‐design methods that can support the robust and rigorous implementation of these methods (for example, pre‐registering whether co‐production or co‐design will be utilised). Such improvements will be necessary as these methods become increasingly mandated by major funders of mental health research. Nevertheless, embedding PPI in research on children and young people's mental health has the potential to significantly improve the quality of research in this area and the benefits to young people experiencing mental health problems.

## AUTHOR CONTRIBUTIONS

**Alex Lloyd**: Conceptualization; investigation; methodology; writing ‐ original draft; writing ‐ review & editing. **Tom (Chin‐Han) Wu**: Formal analysis; writing ‐ review & editing. **Laura Lucas**: Formal analysis; project administration; writing ‐ review & editing. **Adeola Agunbiade**: Writing ‐ original draft. **Romana Saleh**: Writing ‐ original draft. **Pasco Fearon**: Conceptualization; methodology; supervision; writing ‐ review & editing. **Essi Viding**: Conceptualization; methodology; supervision; writing ‐ review & editing.

## CONFLICT OF INTEREST STATEMENT

The authors have declared that they have no competing or potential conflicts of interest.

## ETHICS CONSIDERATIONS

Not applicable to this research review.

## Supporting information

Supplementary Material

## Data Availability

Data sharing is not applicable to this article as no new data were created or analyzed in this study.
