# Supplementary material for: No decision about me, without me: Collaborating with young people in mental health research
Source: JCPP Adv. 2024 Dec 11;4(4):e12291. doi: 10.1002/jcv2.12291 (PMC11669772; doi:10.1002/jcv2.12291)
Supplement: Supplementary file 1 — Supplementary Material [file JCV2-4-e12291-s001.docx]

**Supplementary Information**

**Study Authorship**

As reported elsewhere (Bakermans-Kranenburg & Ijzendoorn, 2024), there was variation in the included studies with regards to whether experts by experience were listed as co-authors on journal articles. Eight studies listed experts by experience as co-authors, meaning the majority of studies (29) did not include experts by experience as co-authors. One study listed experts by experience in the Acknowledgements of their paper (Culbong et al., 2022).

**Additional Demographic Information**

Only 13 of 37 studies reported the age of experts by experience (14.8 years on average). Twenty-one studies included descriptive information about the gender distribution of experts by experience, with 56.05% of participants across these studies identifying as female. Eleven studies reported the ethnicity of experts by experience, with nine studies having a majority of White individuals in their co-production or co-design activities (Bennet et al., 2022; Cheng et al., 2021; Hill et al., 2022; Hugh-Jones et al., 2022; Li et al., 2022; Mindel et al., 2022; Povey et al., 2022; Thomson et al., 2022; Thorn et al., 2020; Stoyanov et al., 2021). Only four studies reported information relevant to the experts by experience’s socioeconomic status (Bennett et al., 2021; Gobat et al., 2021; Li et al., 2022; Thomson et al., 2022). A common limitation identified in many studies (*N*=24) was that the group of experts by experience involved in the research were not representative of the wider population (Björling et al., 2019; Brooks et al., 2021; Cheng et al., 2021; Davison et al., 2022; Hackett et al., 2018; Hill et al., 2022; Gabrielli et al., 2020; Gobat et al., 2021; Gonsalves et al., 2019; Grové, 2021; Latif et al., 2017; Li et al., 2022; Libon et al., 2023; Mindel et al., 2022; Moltrecht et al., 2022; Morote et al., 2022; Neill et al., 2022; O’Brien et al., 2022; Povey et al., 2020; Stoyanov et al., 2021; Thomson et al., 2022; Thorn et al., 2020; Warne et al., 2022; Ziecshank et al., 2021).

**Total Number of Studies Reporting PPI Activities**

To consider the prevalence of PPI, including co-production and co-design, in developmental psychopathology research, we examined the number of studies in our review that reported using PPI methods irrespective of whether these studies reported demographic information or described the PPI activities conducted. The total number of studies that reported any PPI was 54. Given the total number of records screened (2130), the proportion of studies reporting any PPI activities was 2.54%. While this figure was slightly higher than previous research (Sellars et al., 2020), we note Sellars and colleagues reviewed health studies not limited to *mental* health. Further, our search term was designed specifically to identify studies using PPI methods. Together, these factors suggest that the prevalence of PPI in child and adolescent mental health research is relatively low as a proportion of the total number of studies conducted in this area.

**Infographic Outlining Guidance for Young Advisors**


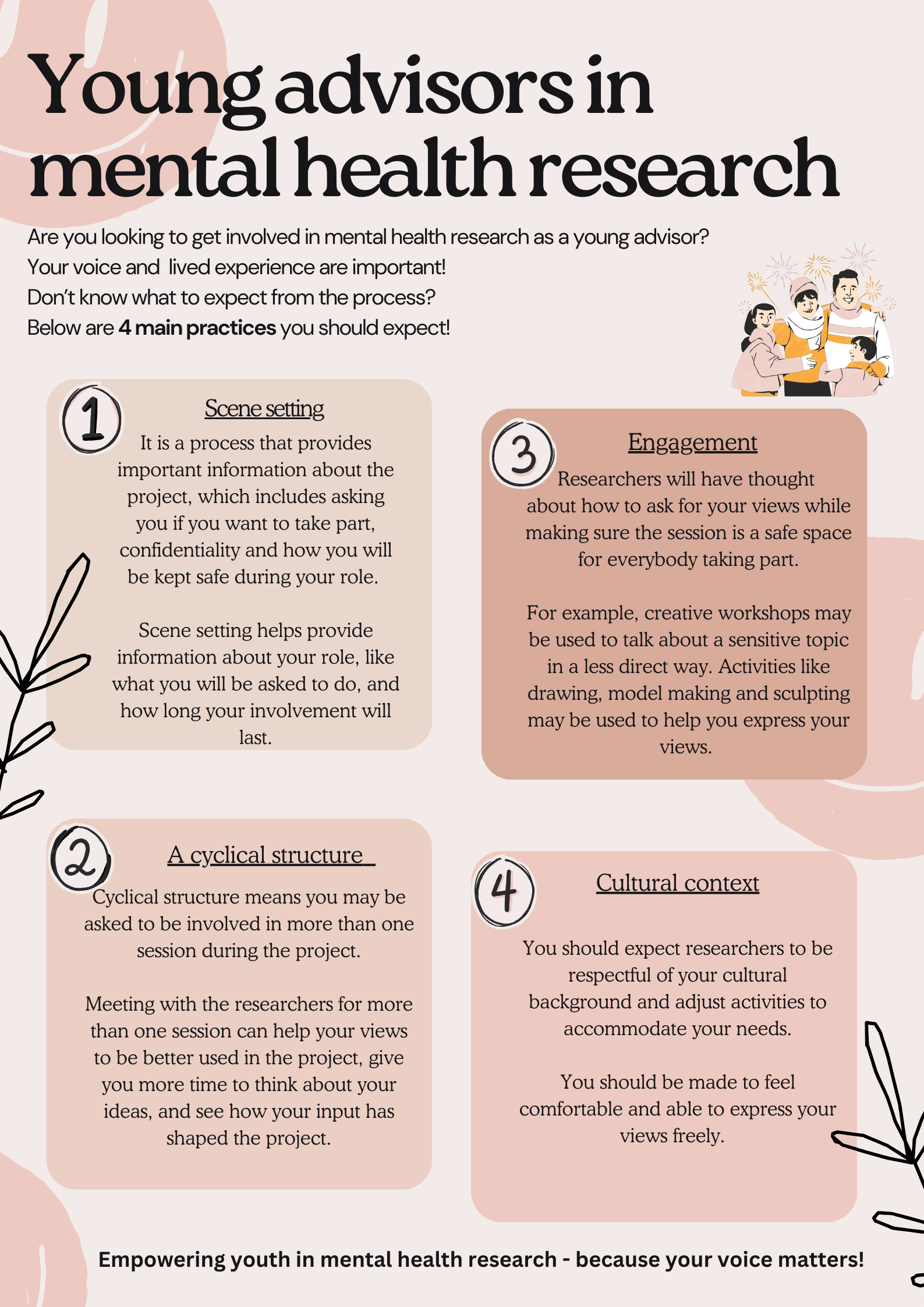


**Supplementary Table 1: Description of included studies**

| **No.** | **First author** | **Year** | **Country** | **Recruitment** | **Topic** | **Sample size** | **Age (M = mean, R = range)** | **Gender (%f)** | **Ethnicity** | **Socio-economic info** | **Training and supervision** | **Remuneration** | **PPI Method** | **PPI Activity** | **No. of sessions** | **Stage of research collaborated** | **Output** |
| --- | --- | --- | --- | --- | --- | --- | --- | --- | --- | --- | --- | --- | --- | --- | --- | --- | --- |
| 1 | Abel | 2020 | UK | NHS sites | General mental health | 14 | R = 12-16 | NR | NR | NR | NA | NA | Co-production | Interviews and focus groups | 1 | Design | Communicated to stakeholders and impacted structure of intervention |
| 2 | Bennett | 2022a | UK | Through existing network | Trauma and abuse | 10 | R = 14-18 | 80 | 100% White British |  | Training on qualitative analysis, scaffolding for existing skills. Background information summarised and provided | £10-15 | Co-production | Workshops to 1) discuss inclusion and exclusion criteria, 2) analyse data, and 3) interpret data and themes | 3 | Design, analysis and interpretation | Determined search criteria, helped to identify themes, supported dissemination of work |
| 3 | Bennett | 2022b | UK | Through existing group of young people involved in research | Help-seeking | 10 | R = 14-18 | NR | NR | NR | Trained in analysis method and background to topic | NA | Co-production | Developed sampling strategy, inclusion/exclusion criteria, searched for eligible studies, supported analysis and interpretation of data | 4 | Design, analysis, interpretation | Informed findings of the project |
| 4 | Björling | 2019 | USA | Event in local library | Anxiety and depression | 114 | R = 13-18 | NR | NR | NR | NA | NA | Co-design | 1) Workshop, stressor/calmer board 2) group interview to storyboard 3) workshop to explore environment 4) workshop to explore nature trek | 4 | Design | Stimuli development, storyboarding for the app |
| 5 | Brooks | 2021a | Indonesia | Via primary care services, third sector organisations, schools and CYP health services | Mental health literacy | 43 (interviews), 13 (workshop) | R = 11-15 | NR | NR | NR | NA | Travel expenses | Co-production | Semi-structured interviews and then 1-day workshop involving creative activities to determine preferences for content, format, delivery, promotion and implementation of intervention. | 2 | Design | Specified why intervention was needed, content of intervention, format and setting of intervention and mode of delivery. |
| 6 | Brooks | 2021b | UK | Existing networks | Mental health in neurodisability | 9 | R= 10-17 | 5 female (55.55%) | NR | NR | NA | NR | Co-design | Facilitated meetings using collage, drawing and games to promote discussion | 3 | Feedback | Provided feedback on different interventions identified through rapid review and which features would be most acceptable to young people with neurodisability |
| 7 | Cheng | 2021 | Australia | Via primary youth mental health services | Mental healthcare | 105 |  | 55.24 | 8.57% Aboriginal or Torres Strait Islander | NR | NA | $30AUD | Co-design | Workshops to facilitate discussions around general technology use, technology for mental health. Workshop to evaluate components of intervention. Further workshops to feedback on ideas to supplement prototype, | 10 | Feedback | Identified themes about service delivery which was used to inform how services implement digital mental health tool |
| 8 | Christiea | 2019 | New Zealand | Schools | Digital intervention | 30 | NR | NR | Māori and Pacific youth | NR | NA | NR | Co-design | Interviews,  usability testing | 2 | Design | Refined aesthetic and content |
| 9 | Culbong | 2022 | Australia | Existing contacts and community events | Mental health service provision | 8 | R = 16-24 | NR | 5 Nyoongar young people, two young people from other aboriginal groups, 1 young person both aboriginal and Torres strait islander | NR | NA | NA | Co-design | Workshops involving group yarns to prompt discussions about mental health provision | >1 | Evaluation | Developed model of working practices with aboriginal young people |
| 10 | Davison | 2022 | Ireland | Via specialist schools | Wellbeing (psychometric study) | 35 | M = 14.19 | 48.57 | NR | NR | NA | NA | Co-design | Workshop 1, introduction to project and definition of key terms, workshop 2 & 3, checked understanding of items and associated images, workshop 4, feedback on modified items and discussion of modes of administration, workshop 5, feedback on pilot of measure | 5 | Design and feedback | Changed wording of items, changed tense of items, introduced pictorial prompts, changed response options |
| 11 | Edridge | 2018 | UK | Mental health services or charities | Mental health service provision | 14 | R = 11-19 | NR | NR | NR | NR | Travel expenses and a payment (amount unreported) | Co-design | 1) Workshop: facilitated discussions. 2) interviews | NR | Design | Informed design of mental health application |
| 12 | Gabrielli | 2020 | Italy | School | General mental health | 20 | R = 14-15 | NR | NR | NR | NA | NA | Co-design | Workshop to elicit views on chatbot look and feel, content, duration of the session and suggested improvements | 1 | Design | Entire intervention developed based on feedback from young person |
| 13 | Gellatly | 2019 | UK | Via NHS staff | Mental health of those with a parent with serious mental illness | 14 | M = 11 | 64 | NR | NR | NA | Travel expenses and refreshments | Co-production | Focus group and interviews. Creative methods (e.g., used of emojis or pens to draw) were used to enhance engagement | 4 | Design and feedback | Identified the needs of young people and make changes to the intervention to meet these needs, such as the mode of delivery |
| 14 | Gobat | 2021 | UK | Via schools | Whole-school approaches to mental health | 22 | R = 11-18 | 40.54 | NR | Lower than average free school meal provision at school | NA | NA | Co-production | Focus groups to develop programme theory and logic model, second focus group to confirm the model | 2 | Design | Refined core component of intervention and identified active ingredients. Identified barriers to implementation of intervention and outcomes of interest |
| 15 | Gonsalves | 2019 | India | Schools | Conduct problems, anxiety, depression | 46 (focus groups), 22 (co-design) | M = 14 (focus groups), M = 14 (co-design) | 36.76 | NR | NR | NA | NA | Co-design | Focus groups regarding use and preferences for different digital media, views on appropriateness of digital mental health intervention in school setting. Co-design workshop on exploring features of popular apps, paper prototyping, discussion about prototype. | 2 | Design | Checked understanding of the app and ensured it was culturally appropriate. Identified format and structure of app that would be most appealing, as well as most common issues faced by this population. |
| 16 | Grové | 2021 | Australia | NR | Depression and anxiety | 40 (questionnaire), 15 (interview) | M = 16.8 (questionnaire), M = 16.2 (interview) | 70.83 | NR | NR | NA | $10AUD | Co-design | Questionnaire asking young people about what they need from online 19support. Interviews elicited views about what they found helpful in digital applications. | 1 | Design | Identified key themes young people sought in a the chatbot |
| 17 | Hackett | 2018 | Canada | Through mental health services | Mental health service provision | 19 (interviews), 12 (app feedback), 5 (co-design event) | R = 16-24 | NR | NR | NR | NA | NA | Co-design | Semi-structured interviews, co-design workshop to validate experience maps and feedback on prototype development | 3 | Design | Identified points at which young people come into contact with services, limitations of services, and how services might be improved |
| 18 | Hill | 2022 | UK | Local child and adolescent mental health services | Anxiety | 4 (phase 1), 4 (phase 2) | M = 10.25 (phase 1), M = 9.50 (phase 2) | 50 | 75% White British, 25% Mixed White and Black African | NR | NA | NA | Co-deisgn | Workshop 1, introduction to project, workshop 2, feedback on intervention stimuli, workshop 3, feedback on initial design including challenges and useability. Phase 2 involved semi-structured interviews | 3 | Design and feedback | Altered design of app to be more appealing and intuitive to children |
| 19 | Hugh-Jones | 2022 | UK | NR | Mental health support in schools | 31 | R = 14-19 | 38.71% | 1 British Afghan, 1 Pakistani, 1 Russian, 1 Spanish, 1 Indian, 1 Black African, 25 White British | NR | NA | £20 | Co-design | Workshops to identify, define, position and concept, workshops to improve initial design of application, workshop to user test complete app and make final changes. | 8 | Design | Identified priorities for adolescents using the application, informed the design and content of the application, approved final version of the application. |
| 20 | Hugh-Jones | 2023 | UK | Local secondary schools | Virtual reality intervention | 17 | R = 15-18 | NR | NR | NR | NA | NA | Co-design | Feedback on priority setting activity, endorsement of intervention approach, individual feedback on intervention stimuli and design. | 2 | Design | Changed design of the intervention, agreed priorities when designing the intervention, directed the design of the VR environment |
| 21 | Latif | 2017 | UK | Through CAMHS | Self-harm | 10-18 | M = 15 | 100 | NR | NR | NA | £20 voucher | Co-production | Workshop including storyboarding activity and discussions | 1 | Evaluation | Contributed content to educational programme |
| 22 | Li | 2022 | Australia | Social media | Depression and anxiety | 36 | M = 14.94 | 63.9 | 5.6% aboriginal or Torres Strait islander, 83% born in Australia | 94.4% owned a personal smartphone | NA | $30 AUD | Co-design | Focus group to develop and feedback on design | 3 | Design and evaluation | Contributed to the design, stimuli and content of intervention |
| 23 | Libon | 2023 | Canada | Community partners and email listservs | Suicide prevention | 11 | R = 15-24 | NR | NR | NR | NA | NA | Co-design | Workshops to elicit views on topic, review challenges surrounding the programme, and consider how to implement ideas | 3 | Design | Contributed to the model of intervention utilised |
| 24 | Mindel | 2022 | UK | Charity | Digital mental health service | 11 | R = 16-25 | 55 | 55% White, 18% Asian, 9% Black, 18% Mixed Ethnicity | NR | Charity training | NA | Co-design | 1) Workshops to define key terms, identify underrepresented groups. 2) Workshop to review initial findings, 3) Workshop to discuss how to translate findings into media accessible to young people | 3 | Design, analysis | Defined key terms, informed analysis, informed dissemination |
| 25 | Moltrecht | 2022 | UK | Through existing networks | Emotion regulation and mental health | 21 | R = 12-19 | 50 | NR | NR | Training prior to project | Not specified | Co-design | Workshop 1, facilitated discussion about mental health, emotion regulation, how technology can be used to support mental health, perceived barriers and facilitators to the use of mental health apps, workshops 2 and 3, feedback on potential designs of app and generated ideas for improvements to features | 3 | Design | Developed a list of do’s and don’ts for mental health apps, edited content of app and introduction of feature to provide immediate support |
| 26 | Morote | 2022 | Spain, Italy, Poland, Denmark, and Iceland | Schools | General mental health | 89 | R = 12-14 | 77 | NR | NR | NA | NA | Co-design | Workshops including discussions, feedback on initial design of intervention | 1 | Design | Adapted intervention to specific locations or regions |
| 27 | Neill | 2022 | Northern Ireland | Via schools | General anxiety and test anxiety | 32 | R = 12-18 | 0 | NR | NR | NA | NA | Co-production | Focus groups conducted to discuss questions related to mental health issues in young people and possible intervention components | 1 | Design and feedback | Fed back on the delivery of the intervention and specific components |
| 28 | O’Brien | 2022 | Australia | Online advert | Eating disorders | 5 | M = 16.6 | 100 | NR | NR | NA | $35 Amazon voucher | Co-design | Semi-structured focus group | 2 | Design | Changed features of programme to make it more appealing |
| 29 | Povey | 2020 | Australia | School or mental health service | Digital intervention | 45 | M = 14.71 | 47 | NR | NR | Training on confidentiality and safety | NA | Co-design | Workshops: discussion of vignettes, body mapping exercise, familiarization with resources, review and discussion of resources | 6 | Design | Identified engaging content & integrated into application |
| 30 | Povey | 2022 | Australia | Schools, drug rehabilitation facility, social media advertisement | Mental health app development | 75 | 15.14 | 53 | 21% English not main language spoken at home | NR | NA | Not specified | Co-design | Workshops held to design and refine app, including storyboards and app prototypes | 21 | Design and feedback | Led to specific design features of the app, including customization, improved storytelling, ensuring option of indigenous language was available. |
| 31 | Realpe | 2020 | UK | Through local partners (charity and academic) | Intervention (psychosis) | 20 | R = 16-25 | NR | NR | NR | NA | NA | Co-design | Workshop to develop initial ideas, workshop to prototype virtual world, workshops to screen prototype, workshop to beta test virtual environment and workshop to pilot intervention | 5 | Design, development, screening, testing and feedback | Integrated feedback to make the app engaging and consider barriers or facilitators |
| 32 | Syed Sheriff | 2022 | UK | Social media | Online intervention development | 12 | R = 16-24 | NR | NR | NR | NA | NA | Co-production | Workshops focusing on intervention development, feedback on stimuli, important themes | 5 | Design | Views shaped the intervention development |
| 33 | Stoyanov | 2021 | Australia | Facebook and existing network | Mental health support | 21 | R = 12-19 | 71.42 | 57.14% White Australian, 4.76% New Zeland, 4.76% European, 23.81% Asian, 9.52% Middle Eastern | NR | NA | £20AUD | Co-design | Workshops to select stimuli associated with key terms, facilitated discussions to consider features of technology that would be beneficial for the app, pair work to consider features of ideal app | 3 | Design | Determined name of the app and the concept design of the app. |
| 34 | Thomson | 2022 | UK | Through CAMHS, schools, colleges and charities | General mental health | 100 | R = 16-24 | NR | Majority White | Middle-class | NA | NA | Co-production | After researchers have provided summary of topic, young people discuss the project | 20 | Variable | NR |
| 35 | Thorn | 2020 | Australia | Youth advocacy organisation | Suicide prevention | 134 | M = 21.23 | 53.4 | 9.9% Aboriginal or Torres Strait Islander | NR | NA | NA | Co-design | Workshops to feedback on design or user-testing of intervention. | 11 | Design | Fed back on content of media campaign to ensure focus was on positives, advocated for representation in the campaign |
| 36 | Warne | 2022 | UK | Via schools | Genes and mental health | 11 (activity 1) and 12 (activity 2) | R = 14-17 (activity 1) and R = 10-13 (activity 2) | 27.73 | NR | NR | NA | NA | Co-production | Facilitated discussion regarding research topic | 1 | Design | Changed study protocol, developed workshop to disseminate findings |
| 37 | Zieschank | 2021 | Australia | Social media | General mental health | 62 | M = 8.1 | 50 | NR | NR | NA | $20AUD | Co-design | Interviews | 3 | Design | Edited stimuli design |

NR = Not reported

Reference:

Warne, N., Rook, S., Jones, R. B., Brwon, R., Bates,L., Hopkins-Jones, L., Hall, J., Langley, K., Thapar, A., Walters, J., Murphy, S., Moore, G., Rice, F., & Collishaw, S. (2022). Collecting genetic samples and linked mental health data from adolescents in schools: Protocol coproduction and a mixed-methods pilot of feasibility and acceptability. BMJ Open, 12(2), e049283. https://doi.org/10.1136/bmjopen-2021-049283
